# A Network Control Theory Approach to Modeling and Optimal Control of Zoonoses: Case Study of Brucellosis Transmission in Sub-Saharan Africa

**DOI:** 10.1371/journal.pntd.0001259

**Published:** 2011-10-11

**Authors:** Sandip Roy, Terry F. McElwain, Yan Wan

**Affiliations:** 1 School of Electrical Engineering and Computer Science, Washington State University, Pullman, Washington, United States of America; 2 School for Global Animal Health, Washington State University, Pullman, Washington, United States of America; 3 Department of Electrical Engineering, University of North Texas, Denton, Texas, United States of America; Universidad de Buenos Aires, Argentina

## Abstract

**Background:**

Developing control policies for zoonotic diseases is challenging, both because of the complex spread dynamics exhibited by these diseases, and because of the need for implementing complex multi-species surveillance and control efforts using limited resources. Mathematical models, and in particular network models, of disease spread are promising as tools for control-policy design, because they can provide comprehensive quantitative representations of disease transmission.

**Methodology/Principal Findings:**

A layered dynamical network model for the transmission and control of zoonotic diseases is introduced as a tool for analyzing disease spread and designing cost-effective surveillance and control. The model development is achieved using brucellosis transmission among wildlife, cattle herds, and human sub-populations in an agricultural system as a case study. Precisely, a model that tracks infection counts in interacting animal herds of multiple species (e.g., cattle herds and groups of wildlife for brucellosis) and in human subpopulations is introduced. The model is then abstracted to a form that permits comprehensive targeted design of multiple control capabilities as well as model identification from data. Next, techniques are developed for such quantitative design of control policies (that are directed to both the animal and human populations), and for model identification from snapshot and time-course data, by drawing on recent results in the network control community.

**Conclusions/Significance:**

The modeling approach is shown to provide quantitative insight into comprehensive control policies for zoonotic diseases, and in turn to permit policy design for mitigation of these diseases. For the brucellosis-transmission example in particular, numerous insights are obtained regarding the optimal distribution of resources among available control capabilities (e.g., vaccination, surveillance and culling, pasteurization of milk) and points in the spread network (e.g., transhumance vs. sedentary herds). In addition, a preliminary identification of the network model for brucellosis is achieved using historical data, and the robustness of the obtained model is demonstrated. As a whole, our results indicate that network modeling can aid in designing control policies for zoonotic diseases.

## Author Summary

Zoonotic diseases (ones that infect both animals and humans) exact a significant economic and human cost, especially in developing economies. Developing effective policies for mitigating zoonotic infections is often challenging, both because of the complexity of their spread and because very limited resources must be allocated among a range of control options. It is increasingly becoming clear that mathematical modeling, and in particular network modeling, of disease spread can aid in analyzing and mitigating these spreads. Here, we develop a network model for the spread and control of a zoonotic infection, focusing particularly on a case study of brucellosis transmission and control among wildlife, cattle herds, and human subpopulations in an agricultural community. After motivating and formulating the model, we introduce tools for 1) parameterization of the model from time-course and snapshot data, 2) simulation and analysis of the model, and 3) optimal design of control policies using the model. The study shows that the network model can inform design of heterogeneous control policies that mitigate zoonotic disease spread with limited resources.

## Introduction

Zoonoses–infectious diseases that can be transmitted to humans from other animals–incur significant cost though their impact on both agricultural production and human communities. Zoonoses have particular prevalence and impact in the developing world, where low-cost yet effective strategies for their control and eventual eradication are badly needed (e.g., [1], [2]). Control of these zoonoses can be quite challenging, requiring 1) understanding (and sometimes new development) of the surveillance, vaccination, and treatment capabilities of a particular zoonotic agent in human and/or animal populations; 2) recognition/modeling of the mechanisms and rates of spread in each species and between species; 3) cooperation across animal- and public health sectors; and 4) the ability to build the infrastructures needed for control within the limitations imposed by the financial and societal circumstances of the community. Historically, infectious disease specialists in collaboration with governmental organizations have attempted to develop effective control and eradication strategies gradually, using field experience that is unique to the region and disease. A particular challenge in controlling zoonotic infections in this way is to appropriately characterize the animal-human interface that leads to spread, and in turn to appropriately allocate resources in the multi-species system.

Recently, several studies have demonstrated that mathematical modeling can aid practitioners in developing control strategies, by allowing *a priori* comparison of the effectiveness of various control strategies and making explicit the roles played by various species in the spread of the disease (e.g., [3]–[5]). However, efforts to study control of zoonoses using mathematical models remain incomplete. The models used are largely very abstract, often representing animal and/or human populations as a single homogeneous group. Additionally, the current efforts typically only compare a few possible control strategies rather than suggesting a comprehensive design for achieving optimal and robust surveillance and control.

Network modeling of infection spread has been a particular area of burgeoning interest over the last few years, see the articles [6]–[12] for a few representative samples. The network (or, equivalently multi-group- or metapopulation-) modeling paradigm for infectious diseases builds on the classical compartmental models for disease spread in homogeneous populations (e.g., [13]); the network viewpoint was motivated specifically by the recognition that spread patterns are often structured and variable rather than homogeneous, and that control capabilities are targeted. Quite a wide range of analyses have been achieved for network spread models, with a particular focus on understanding the role played by the network's topological structure in its spread dynamics. The network spread models have also served as a context for evaluating practical targeted control schemes [8], [14]. Recently, our group has studied heterogeneous control resource allocation in *multi-group* (network) models for virus spread [14]. This study, as well as analogous efforts on controlling human-engineered networks such as traffic networks [15], show how *targeted controls* can be designed for high performance. That is, via analytical means, they identify certain parts of networks (e.g., certain individuals, sub-populations, or control capabilities) that have disproportionate impact on spread, and so suggest concentration of control resources on these components of the network. While such insights regarding control seems germane to mitigation of zoonoses, our results as yet have only been developed using generic models for transmission and control and have only measured spread in terms of one measure (the basic reproductive number), have not considered surveillance at all.

Models for zoonotic diseases (including for brucellosis) classically have been simple compartmental models rather than network-structured models, with each compartment capturing homogeneous transmission within an entire species, or perhaps for an age group of that species (e.g., [3]–[5]). Very recently, models for zoonotic infections that capture the detailed spatial or community structure of transmission have been proposed (e.g., [10]–[12]). These models have allowed characterization of the role of the community structure in spread, as well as comparison of plausible control strategies (largely via simulation); however, these efforts do not permit systematic analytical design of high-performance control strategies.

The purpose of this study is to give a comprehensive treatment of the modeling, surveillance, and cost-effective control of zoonoses, by bringing to bear and enhancing a network-control-theory approach to virus-spread control. As a specific case study, we explore modeling and design of surveillance and control for brucellosis in a prototypical agricultural setting in a resource-constrained area such as sub-Saharan Africa. To this end, a network model for brucellosis transmission among animal herds and to human populations is developed, that captures the mechanism of transmission of this zoonotic bacterium, allows repesentation of realistic surveillance and control mechanisms and their costs, and measures the performance of the control scheme with regard to the disease's financial and societal impact. Once the model has been formulated in a general way, we discuss approaches for inferring important network-model parameters from limited experimental data, which include both simple heuristic approaches and new network-estimation tools from the control sciences. Using the parametrized models, design of surveillance and control capabilities is pursued, with the aim of suggesting good targeted control/surveillance strategies as well as improvements to existing strategies. A particular focus of the design is to obtain simple insights into high-performance strategies that do not rely on model details, so that the developed strategies are in some measure robust to inaccuracies and limitations in the models and available data.

This study advances the existing efforts on network modeling of infection spread, including our own earlier work, in several ways. First, it makes explicit the grapical modeling of spread in multiple species/breeds using a layered network structure. Second, it carefully models a family of surveillance and control capabilities for zoonoses, and brings to bear a **network control theory** methodology to comprehensively design the spread control capabilities. This network control theory approach is valuable, because it permits systematic design of multiple control and surveillance capabilities in a multi-faceted network, to meet multiple performance criteria or optimize a performance measure. We believe that this comprehensive design capability is of central importance for the zoonotic disease mitigation problems that are studied here, because very limited and heterogeneous control resources must be used in many zoonotic-disease control scenarios.

## Methods

We find most illustrative to introduce the proposed layered-network modeling framework for zoonotic diseases using a case study of brucellosis transmission, both to allow careful illustration of how disease-specific characteristics can be captured using the modeling framework, and to permit development of quantitative control policies for this particular neglected zoonotic disease. To begin, Let us briefly overview the methods for modeling brucellosis spread and designing control strategies. In many communities with high brucellosis prevalence (e.g. in West Africa), transmission dynamics and control capabilities/costs vary significantly from herd to herd, because of variabilities in agricultural system, herd and pasture sizes, accessibility, and financial resources [16], [17]. Thus, a model for transmission at the herd level (that further considers multiple distinct human subpopulations) is promising for informing systematic surveillance and design of targeted control strategies. Specifically, the herd and subpopulation-level contact-network model that we propose is a linearized multi-group model based on population-dynamics concepts (see e.g. [6], [14]). We enhance existing models of this form to 1) differentiate spatial spread characteristics among multiple species and 2) explicitly capture realistic multi-faceted control capabilities and costs as network structure modifications and feedback controls. Based on this formulation, we bring to bear and extend a family of recently-developed methods for structure and controller design in complex dynamical networks [14], [15], to develop optimal policies for allocating heterogeneous resources to mitigate zoonotic disease spread in a way tht exploits community (network) structure. Particular results include designs that are tailored to reflect variabilities in agricultural practices, and ones that optimally trade off infection costs in the human and animal populations. Additionally, we apply *system identification* techniques to infer parameters of the spread model from snapshot and time-course data. In particular, these methods are used to parameterize the brucellosis spread model, using snapshot data on bovine brucellosis in West Africa, and time-course data from the Jackson bison herd.

The section is organized as follows. After a brief overview of the brucellosis case study, we present the nominal network model for brucellosis transmission among multiple animal herds and human subpopulations that was developed. Subsequently, we present the modeling of control efforts and costs. Finally, methods for control design and model identification are discussed briefly.

### Case Study Overview: A Model for Brucellosis Transmission and Control

Brucellosis is a zoonotic bacterium with several species that cause illness in livestock (including cattle, small ruminants, pigs, camels, and bison, see e.g. [16]–[19]), wildlife (including bison, elk, and caribou), and humans. The disease in animals is chronic and impacts the reproductive system, with abortion, reduction in fertility, reduced milk yield, and abscess formation as typical signs (that may be temporary or long-term). Brucellosis may also be transmitted from these species to humans, in whom the disease is manifested in severe intermittent fever and extreme fatigue and malaise over a period of weeks or even months, sometimes progressing to a chronic disease with possibility of relapse and numerous complications (including joint/bone problems, gastrointestinal problems, and abortion, among others). Where the disease is prevalent, it may have significant societal and economic impact due to both reduced yield in livestock agriculture and loss of human life and productivity.

The most common strain of *Brucella* in cattle and various wildlife is *B. abortus*. Transmission of *B. abortus* is primarily through contact with aborted fetuses or, in humans, ingestion of raw products from the livestock (such as unpasteurized milk). Pastoralists are often subject to both means of transmission, while a broader segment of the community in developing countries may be subject to infection from consumption of raw products, see e.g. [1] for details.

### Modeling the Nominal Transmission of Brucellosis

The developed model tracks brucellosis prevalence (numbers of infectives) in individual herds for multiple animal species and prevalence in human subpopulations (divided by susceptibility). Specifically, brucellosis infection is modeled in 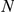 types of animals (i.e. 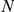 species, or possibly subspecies or breeds if transmission characteristics are different), labeled 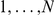, which may include both livestock and wildlife. For species 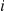, let us assume that 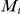 *herds* or groups are present, labeled 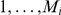. Because the disease is typically chronic in livestock, and dominantly impacts the reproductive system, we believe that considering a single infection class for each herd is sufficient for the initial controller design being pursued here. Specifically, the number of infected individuals in herd 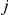 of species 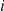 at a time 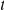, denoted by 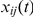 for 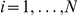, 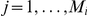, is tracked oer time. The total number of individuals in the herd is assumed constant with the motivation that, over the time horizon of interest, economic and resource-limit determinants typically keep herd sizes relatively stable; the herd size is denoted by 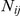. Additionally, the number of people 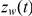 infected with brucellosis is tracked in 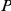 human sub-populations, labeled 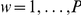, that have different interaction characteristics with livestock.

Here, we first develop a predictive mathematical model for the dynamics of the **infection counts** 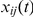 and 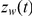, in the nominal case without application of designable controls. Infection-spread dynamics at the scale of herds or sub-populations are often represented using deterministic differential-equation models (known as multi-group models) in the mathematical epidemiology literature [6], [14], and we use and enhance this modeling paradigm here. Given the above-described mechanisms of transmission, it is clear that both transmission between individuals within a herd and transmission among herds (of one or several species) whose members commingle is possible, and we model both means of transmission here. (We note that inter-herd transmission may be especially common for transhumance herds, or ones that share a confined space with other herds in a production system.) Within the herd 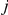 of species 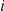, the infection rate due to inter-herd interactions is modeled as proportional to the product of the infected population and the non-infected population, i.e 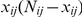; this classical quadratic model is appropriate since the frequency of sexual contact and/or contact with infected birth material (and hence the infection rate) should roughly scale with the pairwise interactions between infectives and non-infected herd members. Even in areas with high brucellosis prevalence, the infected population is typically relatively small (25%) compared to the total population (see e.g. [17]). Thus, the approximation that the non-infected population is approximately equal to the (constant) total herd population 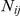 is usually apt. Under these conditions, the rate of infection at time 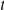 due to transmission within the herd can be modeled by the linear function 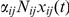, where 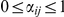 is a breed-specific and herd-specific scaling constant that captures likelihood of spread through interaction (and reflects, for instance, duration of the disease's survival in an aborted fetus, the prevalence of the bacteria in excretion, or the size of the pasture for grazing). Such linear approximations for transmission have been routinely used in network models of spread, and are well-motivated for use in controller design [6], [13], [14]. Here, the linear approximation was used for surveillance and control design, while the nonlinear population-dynamics models was used to verify designs and in identification of model parameters.

Next, the model captures transmission from other herds of the same species. Specifically, consider transmission from a herd 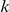 of species 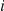 to the herd 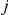 of species 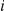, where 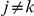. For this pair, the transmission rate is governed by the uninfected population of herd 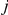 and the infected population of herd 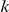, and is modulated by the extent of commingling of the two herds (or, more specifically, interaction of herd 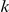
's individuals with herd 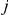 through mixing of the herds or other means, such as purchase of an animal). This extent of interaction is captured in the model using an **interaction scaling parameter** 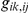, where 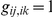 represents identical commingling as would happen within a single herd, and 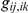 is typically much smaller than 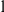. Using the same assumptions on populations that yield a linear model for within-herd tranmission (from 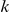 to 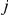), the inter-herd infection rate is modeled as 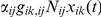. Thus, the total rate of infection for herd 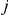 due to transmission from other herds of the same breed is 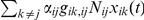.

The rate of infection from herd 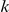 of species 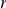 to herd 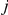 of species 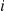 is modeled analogously with the intra-species transmission, but with allowance for varying infectivities. Specifically, the linearized model for this infection rate is 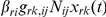. Thus, the total infection rate due to transmission from other species/breeds is 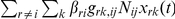.

The model also captures changes in infection counts in a herd due to 1) natural death or (rarely) remission, and 2) incorporation of infected animals from outside the modeled system (e.g., through purchase of the animals by a pastoralist). The rate of decrease in infectives due to death/remission is well-modeled as proportional to the number of infectives in the herd. Specifically, in herd 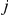 of species 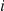, the rate at which infectives decrease through natural death is given by 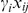 in the model, where 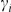 is a species-dependent death/remission rate. Secondly, the infection rate due to injection of infected animals from outside is represented as an *input* 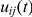 for each herd 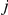 of breed 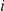. (These additions to a herd are not viewed as changing the size of the herds significantly, but rather reflect e.g. purchase of a few animals to replace losses.)

In the case where the linear model is in force, the following family of differential equations for nominal brucellosis transmission in the livestock/wildlife population is obtained, by summing the transmission rates to each herd and subtracting the natural death rate:
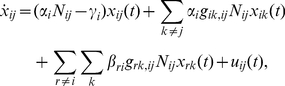
(1)for 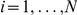 and 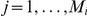. The above model is highly abstracted, in the sense that only one infection state is assumed for each species and the stochastics of transmission are ignored entirely. The model dynamics are in general nonlinear (and we can use the nonlinear approximations as needed), even though our focus has been on the linear approximation with the assumption that the infection fractions are relatively small. This simplistic model for the dynamics is compelling in that it 1) allows systematic controller design and development of simple insights about resource allocation (whereupon a detailed simulation model can be used to test the design) and 2) exposes the role played by the network structure in spread and spread control.

A second core aspect of the nominal model is the representation of brucellosis transmission from livestock to the human population prior to control. As with the animal model, infection of the human population is captured through representation of the infection *rate* at each time, thus yielding differential-equation models of transmission. To roughly capture these infectivity characteristics in a way that permits control design, a linearized population-dynamics model is again used to describe the spread. In particular, each subpopulation is assumed to have an infection rate that is a linear combination of the infective counts in various herds, i.e. the infection rate for human subpopulation 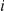 at time 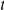 is 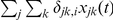, where 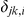 captures the rate of infection caused by herd 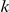 of species 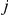. In addition to infection from livestock, the number of infected individuals in each human subpopulation 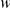 are modeled as declining due to death or remission at time 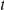, at a rate 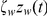. Combining these rates, the number of infected individuals in the human subpopulations are modeled by the following differential equations:
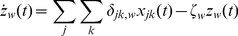
(2)


**Figure 1:**
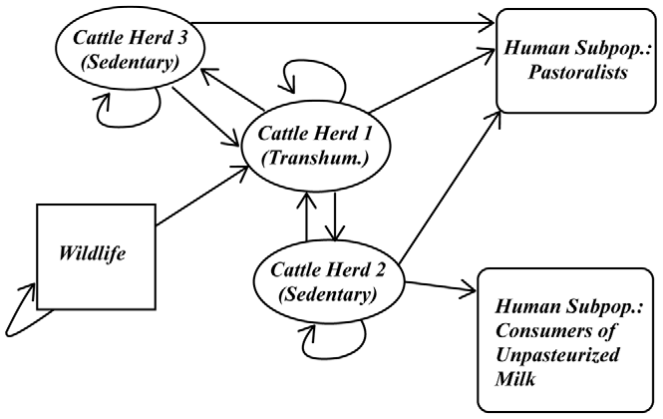
Spread graph illustration. A spread graph for brucellosis transmission is illustrated, for an example with several cattle herds, wildlife that serve as a reservoir for the bacterium, and two human subpopulations.

The nominal model for brucellosis transmission described above can be viewed as a **network model**. In particular, viewing the herds and human subpopulations as *components* in a contact network, we see that there are interactions between pairs of these components if and only if commingling and transmission occur for that pair (with the stength of the interaction reflected in the weight of associated coefficient in the differential equation). To facilitate analysis and design, it is worthwhile to define a weighted and directed **nominal spread graph** or simply **spread graph** that captures the contact network structure. In particular, the spread graph is defined to have 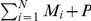 *vertices* or *nodes*, representing each livestock herd and human subpopulation. An arrow or *edge* is drawn from one vertex to another, if and only if the herd/subpopulation corresponding to the first vertex directly impacts the infection rate in the herd/subpopulation corresponding to the second vertex (i.e., if the first herd's infection level is present on the right side of the differential equation for the second herd). The weight of the edge is set equal the corresponding coefficient in the linearized differential equation, since this coefficient captures the strength of the interaction. This formulation of a graph includes *self-loops*, or edges from vertices back to themselves, whose weights are chosen in the same way as for other edges (equal to the coefficient of the herd's infection level in its differential equation). The self-loop weights will be negative, in the case that the rate of remission/death is larger than the spread rate in a herd. The spread graph never has arrows leading out from a vertex representing a human subpopulation, and the self-loops for these vertices are always negatively weighted, since humans do not transmit the bacteria. An illustration of a spread graph is given in Figure 1.

In that the spread graph specifies the contact network (i.e., the transmission rates) among modeled populations, we note that specifying the spread graph accurately for examples of interest is of critical importance. Let us stress that, in general, three different approaches may be used to specify the spread graph: 1) the control-strategy planner may use intimate knowledge of an agricultural system to identify herd contacts and postulate transmission rates; 2) a formal system-identification methodology may be used to infer the spread graph; or 3) for large-scale networks, existing characterizations of typical network topologies (for instance, “small-world topologies” wherein all subunits are within a few steps of each other in the graph despite relatively spare connectivity) can be used to generate plausible spread graphs. In the illustrative example presented in the results, we will motivate and describe the specification of the spread graph that we have used for this example.

### Modeling Control Capabilities and Disease Costs

Next, the nominal multi-group model for brucellosis transmission is enhanced to explicitly represent surveillance and control capabilities 1) within the animal population and 2) for transmission from animals to humans.

In the animal population, control of spread is achieved through several means, including 1) vaccination, 2) surveillance for and culling of diseased animals, 3) limitation of herd commingling (through restriction of trade or movement of livestock), and 4) improvement in sanitation at farms (e.g., [18]). Broadly, these various control capabilities can be viewed as impacting the dynamics of the model in two ways: 1) they alter the rate at which one herd/subpopulation causes brucellosis infection in another herd, or in other words change the model parameters (equivalently, the edge weights in the nominal graph); and 2) they cause removal of infected individuals at particular times, i.e. they serve to change the state variables 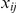 and 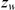. These changes can be captured as modifications and feedback control terms in Equations 1 and 2.

#### Vaccination

Vaccines for brucellosis have been developed for cattle and small ruminants. Application of a vaccine to a herd serves to make the members of the herd less susceptible to the disease. Vaccination capabilities are abstractly incorporated into our model as follows: vaccination of a particular herd 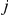 of species 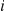 is viewed as scaling all the nominal transmission rates to the herd by a constant 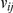 between 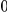 and 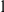. That is, the transmission coefficient from herd 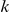 of species 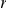 to the herd of interest is changed to 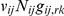, in Equation 1. The constant 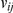, which reflects the effectiveness of the vaccination strategy, in general may be one of several discrete values (with 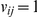 corresponding to no vaccination, and smaller 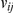 corresponding to stronger vaccination strategies).

#### Surveillance and culling

Surveillance for brucellosis is non-trivial, since the signs of the disease are non-specific. Very broadly, to test for brucellosis with high fidelity requires either identification of the organism cultured from fluid or tissue samples, or identification of infected animals through serological tests; these tests typically trade off specificity and sensitivity, and may be costly. Typically, surveillance is used for control by depopulating livestock that are identified as infected. Thus, a surveillance and culling program is abstractly modeled as one that removes infectives from herds at some rate. Specifically, the rate at which infectives are removed from herd 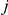 of breed 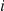 in Equation 1 is modeled as being the nominal rate scaled by a constant 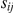 that is larger than 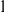, i.e. as 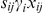. Here, 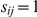 represents the situation that no surveillance/culling is used (so that removal is at the nominal rate, due to death and remission). Meanwhile, larger 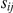 represents an increasingly effective surveillance/culling policy, with arbitrarily large 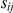 corresponding to perfectly effective and immediate surveillance and culling. As with vaccination, the variables 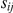 often should be modeled as taking one of several possible values, which represent surveillance/culling programs of different levels of effectiveness. From the network-graph perspective, applying the control in our model serves to change the self-loop weights.

#### Reducing commingling and improving sanitation

Two further methods for mitigating brucellosis spread among livestock are 1) limitation of commingling among herds, and 2) improved sanitation. Like vaccination and surveillance/culling controls, these further control methods can be naturally modeled as altering the nominal model and so the nominal interaction graph in various ways. Specifically, reducing commingling among herds will serve to reduce the interaction weights 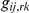 and hence to reduce the transmission rates between herds (or in other words reduce the edge weights between different vertices in the interaction graph). Depending on the manner in which commingling is limited, interaction weights throughout the network may be limited, or only certain tranhumance herds may be affected. Meanwhile, improved sanitation and housing for livestock will reduce the infection rates *within* herds in Equation 1, and hence can be modeled as scaling these infection rates.

#### Controlling Transmission from livestock to humans

Brucellosis is a severe and in many cases incapacitating disease in humans, whose prevention is paramount. No vaccine for brucellosis exists for humans. Instead, transmission from livestock to humans is primarily limited in three ways: 1) pasteurization of milk products and proper preparation of meat products; 2) brucellosis surveillance and control programs; and 3) reduction of transmission to those handling livestock products through improved sanitation and training in safe handling of livestock products. These control methods fundamentally serve to reduce the transmission rates to one or more human subpopulations from some (or possibly all) of the animal herds. That is, the controls serve to scale the nominal rates of transmission 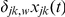 in Equation 2 by weights between 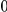 and 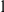. For instance, proper pasteurization of milk will scale the transmission coefficients from all milk-producing herds to populations that traditionally have unpasteurized products, in particular changing the coefficients from 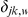 to 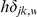 where 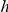 reflects the effectiveness of the pasteurization process. It is worth noting that the methods for preventing transmission to humans, while seemingly basic, may require significant investment in developing countries (e.g., requiring development of a cold chain from source to distribution).

#### Modeling costs and posing the control design problem

Broadly, designing effective control strategies requires achieving a proper tradeoff between the costs resulting from disease prevalence and the costs of control. Altenatively, control design can be viewed as an effort to minimize disease prevalence or associated costs, while using limited control resources. Thus, to properly design control strategies, models are needed for the cost of infection as well as the costs associated with using the various control actions. As a framework for cost modeling, each infected individual in each herd and human subpopulation at each particular time is viewed as incurring a cost, and these costs are summed to obtain the full infection cost. Precisely, the infection cost at a particular time 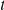 is obtained as 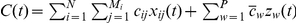, where the weights 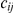 and 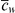 represent the incremental (per-animal) cost of infection for each herd and human subpopulation. It is worth stressing that the incremental cost of infection may be different for each herd and human sub-population, for instance infection of milking cows/sheep may incur greater cost. The total infection cost over a period of time, 
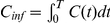
, is often of most interest.

In addition to the costs incurred by brucellosis spread, the cost of control is modeled. For each possible control action (e.g., surveillance using serological tests followed by culling), the cost is assumed to have three parts: 1) a global overhead cost for the infrastructure needed to implement the control strategy, 2) a per-herd (or per- human subpopulation) cost representing the additional cost of providing controls to the herd (which may depend on the size and type of herd), and 3) a cost per infected individual in the herd. For instance, for a testing and culling strategy, there is a fixed cost for developing the laboratory infrastructure for testing, a cost associated with testing each herd, and a cost to the farmer for each culled animal. Finally, the total cost of control is calculated as the sum of the costs for each implemented control action. The total cost of control during a period of time is denoted by 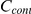.

A control strategy's overall performance is captured by the sum of the infection and control costs over a period of time, 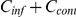, which is denoted as the **full cost** 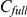. An effective control strategy is one for which the full cost is minimized or at least sufficiently small, over a (finite or infinite) time-horizon of interest. The goal of control design is to achieve such a low-cost or high performance control strategy. Alternately, controls often must be designed to reduce the infection cost, subject to a bound on the cost of control (as specified by the limits on resources available for this specific disease). In either formulation, we stress that achieving a basic reproductive number 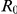 less than 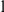 is required in addition to (and usually as a prerequisite for) cost minimization, and this further constraint is systematically observed in our design.

The dynamics of the spread and control model, and associated cost model, are illustrated in the following examples.

### Methods for Analysis, Design, and Parameterization

The layered network model for zoonotic disease transmission that we have introduced above, using brucellosis as a case study, is promising as a tool for systematic design of mulitfaceted control capabilities. Precisely, the formulation permits application of some new methodologies for *network control theory*, for the systematic design of limited control resources to achieve high performance (low spread cost), or equivalently to reduce totaled resource and spread cost. The approach is also promising in that the simple model structure can permit determination of model parameters (parameterization) from sparse historical data, and hence the approach is potentially applicable in the limited-data settings that are common in zoonotic disease spread. In this section, we overview the network-control-theory methodologies for high-performance design and model parameterizaton. We aim to present the methods in sufficient detail that the reader understands the concepts and essential methodologies underlying design/parameterization; to allow wide readership, we exclude technical justifications and algorithmic details, and refer the reader to specific results in the engineering literature for these.

Before describing the methodologies for control design and parameterization, let us stress that the modeling framework permits simple simulation and analysis of the spread dynamics, for a specified control policy. In particular, we note that both the linear and nonlinear differential-equation models can be solved numerically using standard derivative-approximation methods, and can be implemented readily using e.g. the Matlab software. The linearized model also readily permits analysis of the dynamics, including closed-form computation of steady-state and transient dynamics, and computation of features of the dynamics such as the basic reproductive number. This analysis is based directly on the classical analysis of linear systems or linear differential equations; we kindly refer readers who are not familiar with the classical methodology to see [20].

#### Control and surveillance design overview

Control-systems engineers have recently engaged in a major effort to design surveillance and control capabilities in *networks* [21], [22]. This body of controls-engineering research is deeply connected with the “science of networks” that natural scientists have become familiar with [23]–[25], but extends this effort toward control design. Here, we pursue extension and application of the new network control methods to address the brucellosis control problem introduced above, as an illustration more generally of zoonosis-control design.

Previous work has already applied network controller design methods to spread-control problems, albeit for simpler models than the one considered here [14], [26], [27]. The methods used for designing controls for the zoonotic-disease models are similar to those used in previous work. However, the cost measure and set of available control capabilities are more intricate than in previous work, because of the wide range of control capabilities considered and significant differences in spread charcteristcs in the human and animal populatons. The network control theory approach used here combines optimization machinery with graph-theory concepts and a structural understanding of linear systems. Here is a brief description of the procedure:

1. Initially, classical optimization machinery is applied to the design problem. That is, we consider the problem of minimizing the full cost 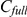 with regard to the design parameters (e.g., 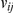, 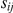) subject to constraints on these parameters. This minimization problem can be resolved using the classical Lagrange-multiplier methodology, whereupon finding the optimal design reduces to solving a system of (nonlinear) equations. It is worth noting that the cost function often is expressed in terms of implicitly-defined functions, most notably eigenvalues of the linearized system's state matrix (e.g., the basic reproductive number). In these cases, computation of the partial derivatives of 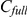 with respect to the design parameters requires us to invoke implicit differentiation methods and in particular *eigenvalue-sensitivity* equations: we kindly ask the reader to see [28] for a review. Once the Lagrangian formulation has been obtained, we progress in two tracks: numerical solution and structural characterization of the optimum.
2. Numerical solution of the Lagrangian can be achieved via numerous standard recursive solvers, such as are available in the Matlab software suite (or can easily be developed by hand). Under certain broad conditions on the network topology and cost definition (which guarantee convexity of the optimization problem), these methods can be shown to obtain a globally optimal design.
3. Often, it is much more instructive to determine structural characteristics of high-performance or optimal designs, rather than to obtain a numerical design. This is especially true of the zoonotic disease control problems considered here, for which socio-political and geographic concerns may make implementation of a precise policy difficult and model parameterization difficult, so that an optimal design may only serve as a guideline rather than an implementable routine. Additionally, by understanding characteristics of high-performance designs, we can make explicit the role played by the network's graph topology in achieving control. For reasons such as these ones, a primary focus of our ongoing work has been to been characterize the system/graph structure of high-performance designs. This structural characterization of high-performance design can be achieved as follows: from the Lagrangian, relationships between the optimal design parameters and the state matrix of the linearized dynamics can be obtained: that is, the dynamics imposes a structure on the optimal resource allocation. Noting that the dynamics of the infection spread are specified using the network graph, we can thus immediately connect the optimal values of the design parameters with the structure of the network graph (e.g., the degrees of the vertices, etc). Further, we can in turn relate the dynamics upon application of the optimal controller to the nominal graph's structure. In this way, we obtain very simple graphical insights into high-performance control designs, that are robust to implementation and modeling limitations. We will present the outcomes of this design methodology in the Results section.

In the interest of conciseness and readability, we have not included many details of the mathematical techniques used and especially their justifications: we kindly ask the reader to see [14], [15], [29], [30] for these specifics.

#### Identification and validation of network models for zoonoses

As we will describe in detail in the Results section, the design methodology suggests that high-performance spread mitigation is highly targeted: particular herds or subpopulations require disproportionate control resources because they disproportionately affect spread (or spread cost), either because of their local dynamics or their network interactions. While the focus here has been on obtaining simple insights into such targeted resource allocations, accurate modeling of zoonotic-agent spread can permit increasingly refined design of spread control strategies, by permitting more accurate characterization of the spread impact of the network components (herds or subpopulations). To obtain accurate models for spread control, additional research is needed to on identifying the model's parameters from data.

We have considered the model-identification task for the brucellosis spread application, from two viewpoints: first, from the viewpoint of identifying a model for a single herd using which a network model can be constructed; and, second, from the viewpoint of directly identifying the full network dynamics or important statistics thereof. In particular, a single-herd model has been parameterized using time-snapshot herd-size and seroprevalence data from several countries in West Africa and the Jackon bison herd, as well as using temporal data from the Jackson Bison herd [31]–[33]. Specifically, the data used for parameterization includes: 1) average prevalence vs. average size for small, medium, and large herds for several districts in West Africa, as well as 2) time-course prevalence and herd-size data over a 10-year period for the Jackson bison herd. This parameterization effort uses rather standard heuristic tools from the field of system identification, see e.g. [34]. We note that these individual-herd models also provide indication of the spread-impact of a herd (though not the specific topological structure of this impact), and so permit us to apply many of the results on spread-control design obtained above. Finally, with regard to full parameterization of a network model, new techniques for network identification can be applied [35], once ample data on brucellosis spread within an agricultural network has been obtained. This full network identification to left to future work.

## Results

We find it most illustrative to present the results of the modeling and control design methodology introduced here, in the context of a specific example of brucellosis spread. Specifically, model dynamics and control design are illustrated in an example that is representative at a small scale of largely agricultural communities with both transhumance and sedentary herding (such as in Ethiopia or the Sudan). Specifically, a non-intensive agricultural system with one breed of cattle, comprising 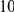 transhumance herds with an average of 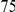 cattle each and 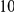 sedentary herds with an average of 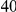 cattle each, is modeled. Farming practices (including cattle density on grazing lands) are assumed to be similar for the herds, and so within-herd transmission rates are modeled as identical: these transmission rates are obtained through model-identification, as detailed below. Further, in this illustrative example, each transhumance herd is assumed to commingle with 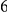 other herds (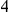 other transhumance herds and 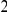 sedentary herds), for a fraction of the year (specifically, 3 months). The human population is subdivided into two subgroups that are subject to infection, 1) pastoralists with a high rate of infection from the cattle and 2) non-pastoralist consumers of raw milk products with a lower but still significant rate. The human-population groups are assumed to be equally impacted by each herd.

### The Nominal Model

**Figure 2:**
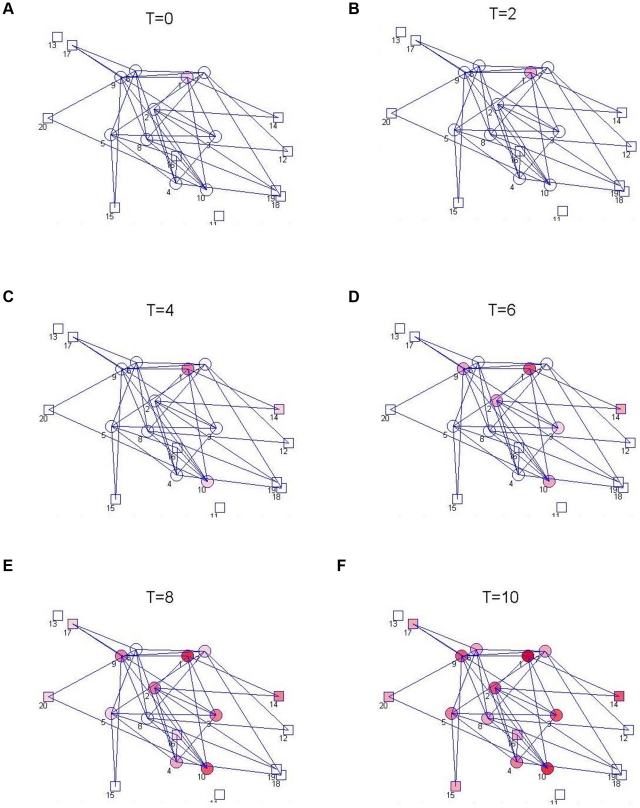
Simulation of nominal spread model from an initial infection in a transhumance herd. Brucellosis among herds of cattle is simulated in a non-intensive agricultural system, in the nominal case that controls are not used. In this small example, a network of 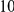 transhumance herds (shown as circles) and 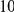 stationary herds (shown as squares) are considered, with varying herd sizes but otherwise comparable intra-herd transmission conditions. Tranhumance herds commingle with other transhumance and sedentary herds, as indicated by the spread graph (which is overlayed on the dynamics). In this example, the possibility for and frequency of spread between herds is specified based on a distance measure between the herds, although other models can be used alternatively. The simulation is initiated with a small number of infected cattle in the largest transhumance herd (Herd 1). The dynamics of the spread with time is shown (with the time axis representing months), with the extent of infection in each herd indicated by the intensity of the red color for that herd. As expected, since the basic reproductive number 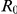 for the spread is greater than 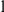, the infection becomes widespread quickly.

**Figure 3:**
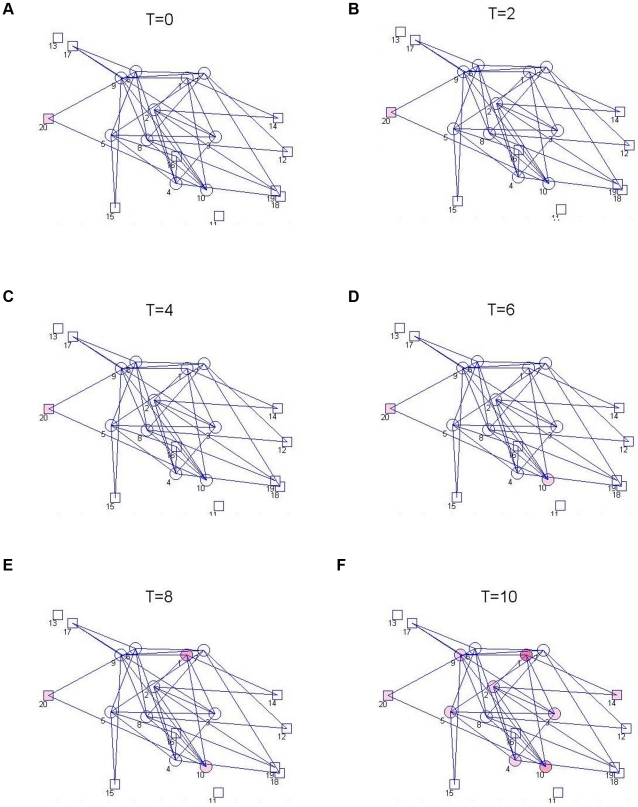
Simulation of nominal spread model from an initial infection in a sedentary herd. The nominal model for brucellosis spread is again simulated for the 20-herd example, without control, but the disease is initiated in a small and sedentary herd (herd 20). The disease again becomes prevalent, but the spread is much slower. Interestingly, brucellosis becomes more prevalent in the large nomadic herds than the small or sedentary ones even though the infection was initiated in a small sedentary herd. This model characteristic matches with field measurements for brucellosis prevalence in pastoralist communities, e.g. in the Tigray region of Ethiopia [17].

The nominal spread graph, which illustrates the contact network prior to control, is shown in Figures 2 and 3. Simulations of spread among the cattle herds are shown for two different initial conditions, one with a single infective in a transhumance herd and the other with a single infective in a sedentary herd (Figures 2 and 3). The basic reproductive number 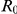 for the nominal model in the animal population is greater than 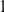, and as expected the infection becomes widespread for both initial conditions. The infection spreads much more rapidly when it initiates in a transhumance herd. Interestingly, even when the infection initiates in a sedentary herd, it eventually becomes more prevalent in the transhumance herds. We note in these examples that we have initiated the infection in the largest sedentary and transhumance herds. We did so because the larger herds display a faster initial growth of the infection, leading to a more rapid (and easier to display) spread in the network. We stress that, qualitatively, the response characteristics would be similar if the infections were initiated in smaller herds, though the progression would be slower. The model also shows that the infection rate in the human subpopulation that is responsible for animal husbandry grows rapidly in the early stages of the infection. A couple remarks about the nominal model are worthwhile.

**Remark 1.** In the 20-herd example chosen above, we note that the *graph topology* of the contact network is one where half of the vertices have high degree, while the remaining vertices have low degree: the graph is generated to enforce a degree distribution, but chosen randomly within this constraint; the particular transmission rates used in the example are obtained through identification of the brucellosis spread model. Such random graphs with enforced low- and high- degree vertices have some properties in common with the common *small-world graphs* when they are of sufficient size, and in this sense our results for the example may be indicative of results when the topology is a small world. We stress, however, that our analysis methods (and subequent control design and identification methods) can be applied *regardless* of the graph topology, and that the specific topology that we have chosen is guided by our understanding of the agricultural practices governing brucellosis spread rather than by the typical model classes considered in the complex-systems literature.

*Remark 2:* In the above example, we have excluded transmission to and from wildlife, because of the difficulty in parameterizing wildlife-related transmission rates. Model simulation and analysis when transmission to wildlife is considered is straightforward. For instance, we have completed simulations in which an identical rate of transmission between each herd and wildlife is assumed: as expected, disease prevalence increases with increasing transmission rate to and from the wildlife reservoir. In practice, we conjecture that the transmission rates between each herd and the wildlife reservoir are better modeled as random quantities, reflecting variability in the extent of contact with wildlife. We leave further development to future work.

### Modeling of Control Strategies

**Figure 4:**
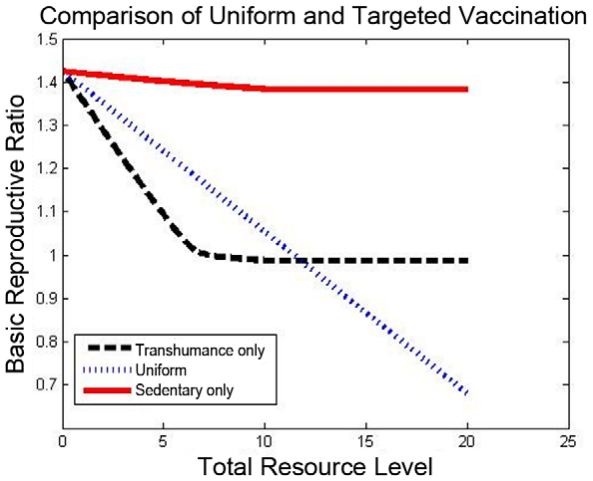
Quantitative comparison of vaccination policies. A uniform vaccination policy is compared with one that targets only the transhumance herds and one that only targets the sedentary herds. At low resource levels, the vaccination policy targeted to the transhumance herds outperforms the uniform one, while the uniform policy becomes more effective at higher resource levels. Both outperform sedentary-herd vaccination.

Several strategies for vaccination, namely ones that are targeted to tranhumance herds vs. ones that distribute resources between tranhumance and sedentary herds, are compared in the 20-herd example. In particular, herds are modeled as being vaccinated at a certain frequency, with the cost needed for each vaccination of a herd assumed identical in this illustrative example. (The relative cost of vaccinating sedentary and transhumance herds may sometimes be rather varied in practice: in some settings, transhumance herds may be incredibly difficult to reach for vaccination, while in other cases medical personnel may be able to take advantage of their mobility by placing vaccination capabilities at a location frequented by these herds. We take the simplest assumption of identical cost for ease in illustration, but the design can be achieved for other cost structures also.) Three strategies with identical total resource cost are compared: one that vaccinates only the transhumance herds, one that vaccinates sedentary and transhumance herds, and one that vaccinates only the sedentary herds. For the three vaccination strategies, the basic reproductive ratio 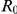, which can be calculated from the spectrum of the *state matrix* of the linearized model dynamics (see [6], [14] for details), is compared. The results are shown in Figure 4. When resource limits are low, vaccination of transhumance herds can reduce the basic reproductive number below 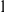 while a uniform vaccination strategy or a sedentary-herd vaccination strategy cannot. When more resources become available, uniform vaccination becomes comparable and eventually preferable to only transhumance-herd vaccination (and reduces 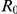 to 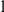). However persistent infections still tend to be common in the tranhumance herds.

**Figure 5:**
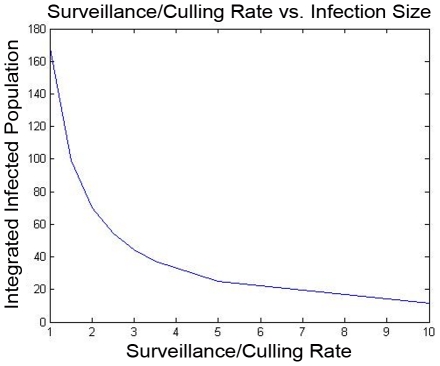
Analysis of a surveillance and control policy. Improvement of brucellosis surveillance procedures so as to permit fast/cheap distributed surveillance and culling is an important policy goal. The model permits computation of infection costs as a function of the surveillance and culling rate, and hence indicates the cost benefit of improving surveillance/culling techniques.

It is hypothesized that improved surveillance techniques that permit fast and cheap decentralized surveillance/culling could significantly improve brucellosis control. The model permits evaluation of the benefits of faster surveillance/culling. Here, a uniform surveillance/culling policy at all herds is considered for the 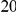-herd example. Specifically, the dependence of an infection cost (specifically, the infected animal population integrated over time) on the surveillance/culling rate is identified. This dependence, shown in Figure 5, indicates the improvement in spread mitigation due to faster surveillance/culling. Using this type of characterization, a practitioner can evaluate whether the additional cost to design new surveillance/culling techniques is worth the improvement in spread cost due to these advances.

Here, we consider a homogeneous surveillance/culling procedure for all herds, and calculate the integrated infection size (the number of infected animals integrated over the duration of the infection) for the 20-herd network, for a random initial condition. In this example, a culling rate of at least 0.43 (43% of the infected population per annum) is needed to make 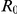 less than 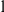; however, higher culling rates beyond this threshold significantly reduce the total infection size.

### Optimized Strategies

**Figure 6:**
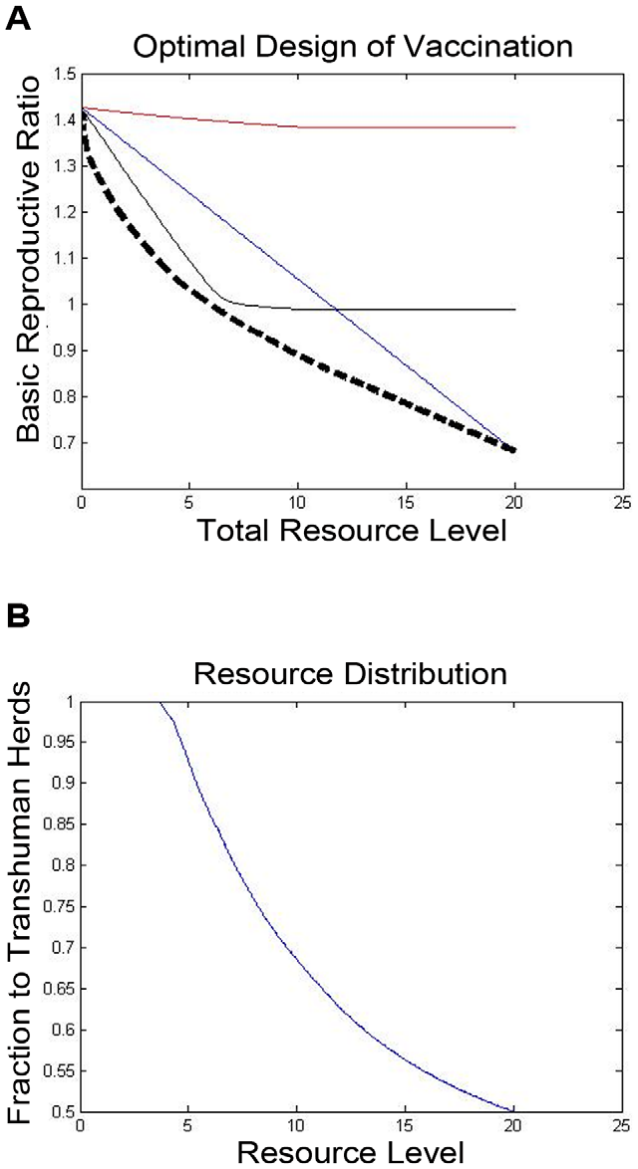
Designing an optimal vaccination policy. In the upper plot, The basic reproductive number when the optimal vaccination policy is used is shown, for the twenty-herd example. Here, the three light, solid lines indicate the performance of the only-transhumance, only-sedentary, and uniform vaccination policies as developed in Figure 4. The performance of the optimal resource allocation is highlighted as a bold, dashed line. Also, the fraction of resources allocated to the sedentary herds at the optimum is shown.

**Figure 7:**
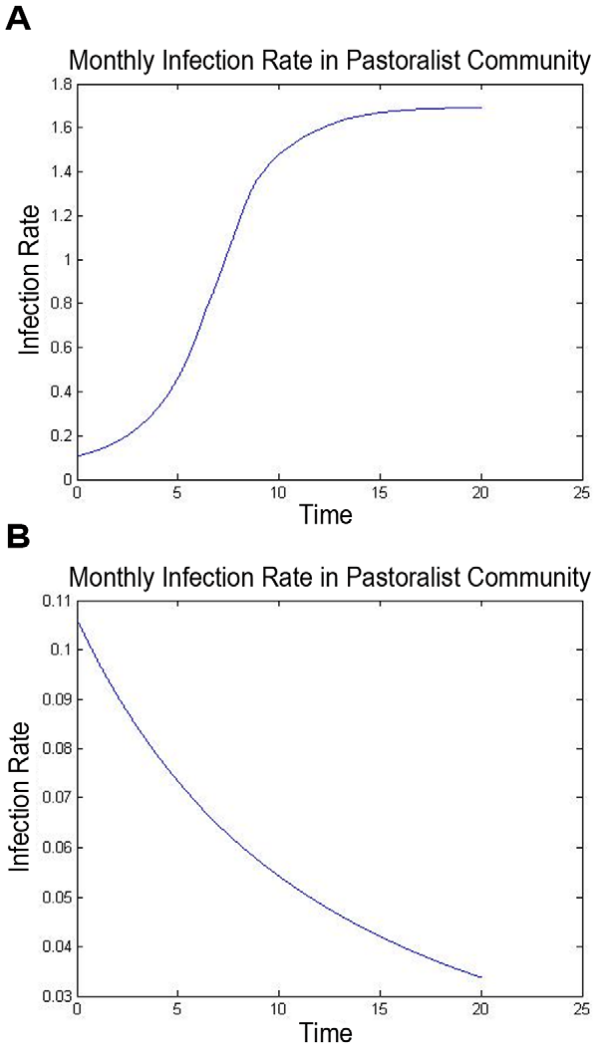
Human infection rate determined by the spread model. The rate of infection in the human pastoralist subpopulation in a brucellosis outbreak with an initial low level of infection in the cattle population is shown. The upper figure shows the case where no control is used, and the lower shows the case where an optimal animal-vaccination policy is applied. It is worth noting that the nonlinear model for spread was used to simulate the infection rate, since the linear model quickly becomes inaccurate in the no-control case.

**Figure 8:**
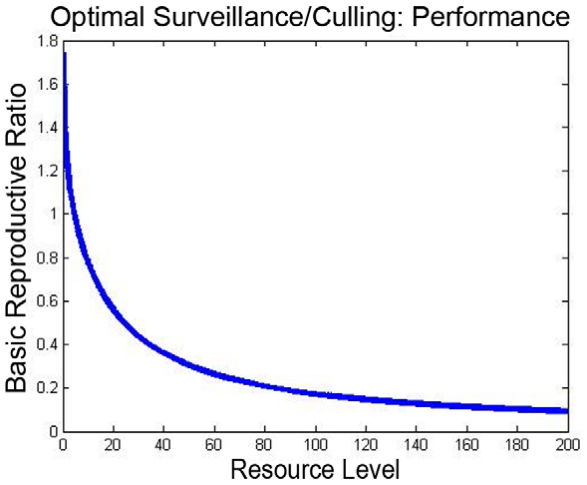
Basic reproductive ratio for optimal design. The basic reproductive number for the optimal surveillance/control policy is shown as a function of the resource level, for the twenty-herd example.

**Figure 9:**
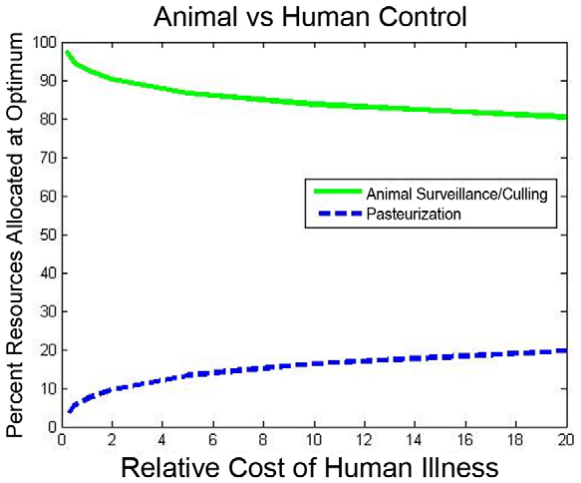
Comparison of animal-level and animal-to-human controls. The subdivision of resources between animal-level control policies and animal-to-human control policies is examined, in the 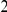-herd example. In particular, assuming a particular relative cost of human infection vs. animal infection (per individual), the optimal division of resources between ainimal surveillance/control and pasteurization is determined. This resource allocation is plotted against the relative cost. If human illness costs are higher, additional resource allocation in pasteurization is beneficial, but the bulk of resources should still be allocated to animal control.

The modeling methodology that we have introduced permits systematic optimization of control resources, according to the methods outlined above. For illustration, we have found the optimal allocation of vaccination resources to minimize the basic reproductive number in the illustrative example, as a function of the level of available resources. The optimal basic reproductive number, and the fraction of the resources that are allocated to the transhumance herds, are plotted in Figure 6. The impact of the vaccination policy on the rate of infection in human pastoralists is also shown (Figure 7). As a comparison with the optimal vaccination strategies (Figure 6), the performance of an optimal surveillance and culling strategy (in terms of the achieved basic reproductive number 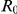) is also shown (Figure 8). Finally, concurrent design of surveillance/vaccination capabilities and pasteurization to prevent transmission to humans is considered in the 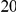-herd network. In this simple example, the costs of vaccinating each herd and of pasteurizing the milk from each herd are all assumed to be identical, and the optimal design (with respect to a total infection size measure) is examined for different human vs. animal infection costs. The results are shown in Figure 9.

### Parameterization

**Figure 10:**
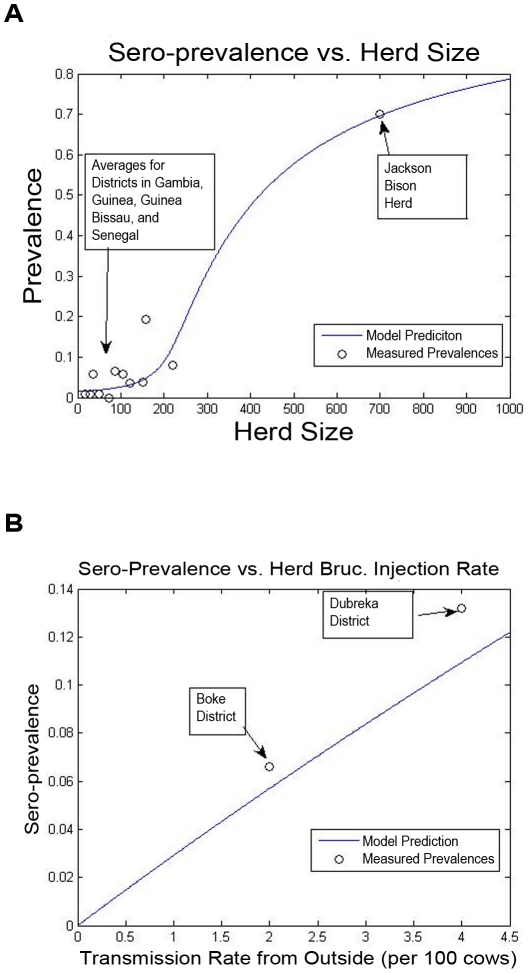
Model identification from snapshot data. Using a heuristic method, a nonlinear SIR model for brucellosis transmission within a herd has been developed, using snapshot herd-size and seroprevalence data from several West-African countries as well as from the Jackson Bison Herd (JBH). The ability of the model to predict seroprevalence vs. herd size is shown *(top)*. Also, for two non-intensive farming districts in Guinea which have similar herd sizes, the amount of inter-herd interaction and hence the comparative rate of outside-herd infection can roughly be guessed, from a description of the prevalent agricultural practices. The model is shown to provide a better indication of prevalence, once this variation is accounted for *(bottom)*.

**Figure 11:**
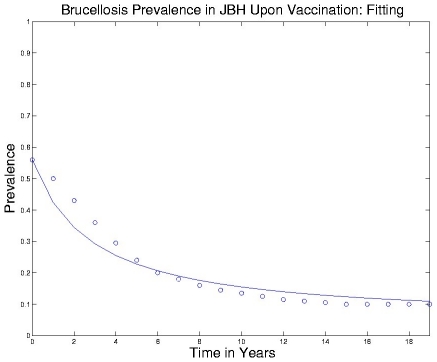
Model identification from time-course data. An SIR model for brucellosis transmission is identified, based on time-course data from the Jackson bison herd upon initiation of a vaccination program. This simple model is not as accurate as the multi-state model described in [31], but is sufficient for the broad policy-design efforts undertaken in this research.

The techniques described above for identification of the bovine brucellosis model from snapshot and time-course described were applied. The results of the identification are displayed in Figures 10 and Figure 11. The identified parameters have been used to specify nominal intra-herd and inter-herd transmission rates in the illustrative example.

## Discussion

We have found it convenient to illustrate the results of our methodology using an example. Let us stress, however, that the methodology has much broader application: the developed network modeling framework can be used to quantitatively capture transmission of a zoonotic disease within a particular agricultural system or community, and in turn can be used for control and surveillance policy design for such transmission. In addition to permitting concrete designs for examples, the tractability of the model also allows us to obtain broad insights into control and surveillance design for brucellosis (and other zoonotic diseases), and conceptual insight into model parameterization from historical data. Let us discuss some broader insights obtained using the modeling methodology, drawing on the example developed in the Results section as needed to make the insights precise. We note that some of these insights admit formal justifications using the model optimization methodology; we omit these mathematical details since they are not central to our development. After this discussion, we also briefly summarize the article.

### Broad Insights into High-Performance Control

The network control methods allow a comprehensive study of the brucellosis controller design problem, and more generally of zooonotic-disease control. Below is a list of several aspects of the control design task that can be addressed using these methods, as well as a few of the key insights obtained through the design. These insights are envisioned as informing policy decision-making.

*1) Optimal or high-performance sub-divisions of control resources among the animal herds can be determined, based on the spread graph's structure.* When resource allocation costs and infection costs due to prevalence in a herd are relatively homogeneous, then high-performance designs tend to equalize the spread-impacts of the herds, to within the extent allowed by the constraints on resources and on the control. Thus, limited resources (whether for vaccination, surveillance/culling, or other purposes) should be allocated to herds with high spread impact (sum of outgoing edge weights from that herd's vertex on the graph), until the spread impacts are equalized. If further resources are available, they should be equally distributed among the herds, if possible. When constraints prevent allocation of resources (or further resources) to some herds, the design becomes somewhat more intricate: some herds must be allocated extra resources so as to limit the spread-impact of their constrained neighbors. These characteristics of resource allocation are indicated in the optimal-vaccination design for the illustrative example, see Figure 7. As expected, the available resources are devoted to the tranhumance herds (which have higher spread impact in this example), until enough resources are available to achieve equal spread impact. The optimal resource allocation significantly outperforms a uniform, transhumance-only, or sedentary-only resource allocation program. Very similar results are also obtained when an integrated infection size measure is used instead (details not shown).

*2) The design method permits comparison of multiple spread-control strategies in the animal population (e.g., vaccination vs. surveillance/culling), and concurrent design of multiple strategies.* For instance, we have pursued a comparison of an optimal vaccination strategy (Figure 6) and an optimal surveillance and culling strategy (Figure 8) for the illustrative example. A careful comparison of multiple strategies (for instance, the two strategies in the example) requires precise knowledge of control and cost parameters, for instance vaccine efficacy and relative-cost information. However, even from simple comparison of Figures 6 and 8, some differences between the two strategies become evident. In particular, one finds that a vaccination strategy can only reduce the basic reproductive number to a particular threshold, while a surveillance and culling strategy can reduce the basic reproductive number arbitrarily near to 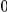, i.e. it can eliminate the infection quickly. Thus, if sufficiently fast reduction of spread is needed (for instance, in a case where the zoonotic infection is especially dangerous to humans and also an efficient transmitter), surveillance and culling will need to be used, albeit perhaps at much higher cost. As cost and effectiveness parameters become available, this tradeoff between vaccination and surveillance/control strategies can be made explicit. Concurrent design of these and other strategies can also be pursued, upon a slight extension of the methodology given in [14].

*3) The design methodology can identify the tradeoff between allocating resources to human population groups rather than to the animal population, as a function of resource and infection costs.* Given the significant heterogeneity in transmission of brucellosis (and other zoonotic diseases) in the human and animal populations, comparing policies that assign control resources to stop transmission among animals with those that prevent transmission to humans is of importance. The newly-developed network design methods permit such comparison, and in turn allow appropriate subdivision of resources for animal-level control and animal-to-human transmission control. As an illustration, let us interpret the concurrent design of surveillance/vaccination capabilities and pasteurization to prevent transmission that we presented for the 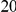-herd example network. In this example, a bulk of the resources are devoted to animal-level control, which prevents infection in both the human and animal populations; however, as expected, the fraction of resources devoted to pasteurization increases as the cost of human illness is assumed to be higher relative to the cost of disease in cattle. The strong benefit of control in the animal popolation is not surprising, since such control effectively reduces disease prevalence in both the animal and human populations. While the specifics of the subdivision will vary with the specifics of the design problem, the design methodology allows for concurrent design of human- and animal- level strategies and characterization of these designs over a range of unknown parameters.

*4) The core resource-allocation designs can be enriched to obtain dynamic or reactive strategies for mitigating zoonotic diseases.* As real-time measurements of trends in infection counts (or costs) become available, strategies that dynamically allocate resources based on these trends can be developed. The reader is referred to [27] for a first effort in this direction. In particular, the article [27] provides a systematic approach to dynamic resource allocation based on use of current and past data, and demonstrates the advantage of such a design over a static allocation in a multi-group SIR example.

### Discussion of Model Parameterization

**Figure 12:**
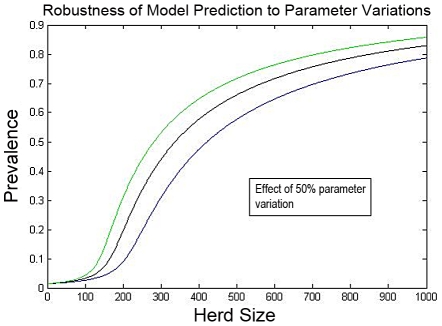
Robustness. Given the very limited and uncertain data available for model parameterization, the robustness of the model to parameter variations is of importance. As a first step in this direction, we have studied the ability of the model to predict single-herd brucellosis prevalence, when there is up to 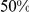 error in each identified model parameter. The above plot shows that the model remains accurate in predicting single-herd brucellosis prevalences despite such variability.

Let us present some insights on the model identification methodology, using the results displayed in Figures 10, 11, and 12 as a context for the discussion:

1. The snapshot data permits characterization of the ratio between the per-individual infection rate and the remission rate (through recovery or death and replacement), for a single herd. As seen in Figure 10, the obtained model provides a reasonably accurate representation of an average-herd-size vs. average-prevalence curve for bovine brucellosis for districts in West Africa and for the Jackson Bison herd.
2. The time-course data further permits inference of the absolute per-individual infection rate, so that (together with the snapshot data-based analysis) a full single-herd model can be identified. Figure 11 demonstrate the ability of the model to describe time-course data for the Jackson bison herd. We note that we have used the parameterized model in the 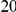-herd example.
3. In parameterizing the single-herd model, we did not have any quantitative data on the rates of infection in each herd from outside the herd: thus, the parameterization has been done assuming an identical rate of interaction/infection from outside the herd. This crude assumption clearly yields error in the parameterization result. For the data from West African herds, we have some qualitative insight into herds that have more significant interactions with other ones (e.g., share common feed lots) and hence are more susceptible to infection from outside the herd. In Figure 10, we also demonstrate that further information on outside infection rates can yield a more accurate parameterization of the model.
4. Given the very limited and highly variable data available for model parameterization, the robustness of the model to parameter variations is of importance. As a first step in this direction, we have studied the ability of the model to predict single-herd brucellosis prevalence, when there is up to 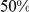 error in each identified model parameter. Figure 11 shows that the model remains accurate in predicting single-herd brucellosis prevalences despite such variability.
5. The uncertainty inherent to the data used for model parameterization highlights the importance of considering stochastics in modeling disease spread. Both intrinsic variability in transmission and uncertainties/variability in model parameters may significantly impact the model dynamics and hence modulate control design (see e.g. [36]). The design approaches that we have pursued display significant robustness to uncertainty (see [14] for details), and so we are confident that the core insights obtained through the design methodology will remain valid even in the presence of uncertainty. Nevertheless, we view enhancing the model to represent uncertainties in dynamics and parameters, and using such models for refined analysis and design, as a critical next step.

### Summary and Conclusions

A network modeling methodology for capturing the spread of zoonotic agents at a herd/subpopulation granularity has been introduced, and used to compare and design control strategies for stopping the spread of zoonoses. The introduced methodology has been developed in detail in the context of a case study, namely modeling and control of brucellosis spread in animal and human populations. Parameter identification of the model from historical data has been pursued.

This modeling and controller design effort should be viewed as a foundational step toward obtaining comprehensive policies for controlling zoonoses from mathematical models: the policies suggested by our methodology must be tested in experimental herds, and the social and political ramifications of control policies and spread mitigation must be considered carefully in defining costs for a zoonosis of interest. It is also important to stress that a wide range of experimental and conceptual methodologies from outside the mathematical-modeling domain must be brought to bear to address policy design for zoonotic diseases, and that mathematical modeling efforts (and policy design more generally) are complementary to core advances in epidemiological methods. Nevertheless, we believe that some promising outcomes have been obtained through this foundational study.

1. Network modeling can give a clear pictorial representation of the spread of a zoonotic disease among subgroups of one or more species. The layered network model that we have developed also permits simulation and simple quantitative analysis of the spread dynamics. Also, a variety of control measures can naturally be captured in the layered network modeling framework.
2. The network modeling approach that we have put forth here also permits systematic and quantitative comprehensive design of resources for control in the animal and human population. Specifically, for the linearized network model studied here, recently-developed tools from the network control theory literature can be applied to design heterogeneous control capabilities to minimize a resource and spread cost. We stress that much work still needs to be done to verify that the abstract controls suggested by the design can be implemented in reality, and can achieve performance similar to that predicted by the model.
3. Network modeling approaches provide sufficient detail to permit comparison and design of heterogeneous control strategies, for instance ones that use surveillance and culling vs. ones that use vaccination, as well as ones that cater to the topology of interactions among the herds. Both qualitative and quantitative predictions and designs can be obtained.
4. Although optimal controller design requires a precise model of spread dynamics and of costs, our design methodology can provide useful insights even when such precise models are unavailable. First, the methodology provides simple insights into good designs–for instance, that more resources should be placed in large and tightly-connected herds–that do not require a precise model to implement. Second, the designs obtained through the methodology show some degree of robustness to variations in model parameters.
5. We have given some preliminary results on parameterizing the network model from data, and have also identified some challenges in fully addressing the parameterization problem. Given the very limited data available on the spread of various zoonotic diseases, model parameterization remains a significant challenge in using methods such as the ones proposed here. Nevertheless, our preliminary efforts on parameterization show promise, and also the model displays a degree of robustness to inaccuracies in parameterization.
6. Clearly, when transmission among small heterogeneous groups is considered (as in our model), stochastics in transmission very significantly impact the spread dynamics. In particular, both intrinsic variabilities in the interactions among individuals that cause spread, and also environmental uncertainties that modify transmission patterns, can critically impact the spread dynamics. Not surprisingly, prevalences of zoonotic diseases such as brucellosis show a large herd-to-herd variability, that can only be explained by considering stochastics in transmission. We have chosen to exclude consideration of stochastics in transmission in this first design effort, 1) because of the great difficulty in parameterizing such stochastic models with limited data, and 2) because our key focus here is on gaining very simple insights into policy design for which even the deterministic multi-group model may be sufficient. We consider the development of stochastic models for transmission to be an outstanding research task of critical importance.

## References

[1] (2006).

[2] Zinsstag J, Schelling E, Roth F, Bonfou D, de Savigny D (2007). Human benefits of animal interventions for zoonosis control.. Emerg Infect Diseases.

[3] Roth F, Zinsstag J, Orkhon D, Chimed-Ochir G, Hutton G (2003). Human health benefits from livestock vaccination for brucellosis: case study.. Bull World Health Org.

[4] Zinsstag J, Roth F, Orkhon D, Chimed-Ochir G, Nansalmaa N (2005). A model of animal-human brucellosis transmission in Mongolia.. Preventive Veterinary Medicine.

[5] Gonzalez-Guzman J, Naulin R (1994). Analysis of a model of bovine brucellosis using singular perturbations.. Jour Math Biol.

[6] Riley S, Frasier C, Donnelly CA (2003). Transmission dynamics of the etiological agent of SARS in Hong Kong: impact of public health interventions.. Science.

[7] Salathe M, Jones J (2010). Dynamics and control of diseases with community structure.. PLoS Comp Bio.

[8] Adams B, Kapan DD (2010). Man bites mosquito: the contribution of human movement to vector-borne disease dynamics.. PLoS One.

[9] Keeling MJ, Rohani P (2008). Modeling Infectious Diseases in Humans and Animals..

[10] Grassly NC, Fraser C (2008). Mathematical models of infectious disease transmission.. Nat Rev Microb.

[11] Galvani AP, May RM (2005). Epidemiology: dimensions of superspreading.. Nat.

[12] Kilpatrick AM, Daszak P, Jones MJ, Marra PP, Kramer LD (2006). Host heterogeneity dominates West Nile virus transmission.. Proc Roy Soc B.

[13] Diekmann O, Heesterbeck JAP, Metz JAJ (1990). On the definition and the computation of the basic reproduction ratio *R*
_0_ in models for infectious diseases in heterogeneous populations.. Jour Math Biol.

[14] Wan Y, Roy S, Saberi A (2008). Designing spatially-heterogeneous strategies for control of virus spread.. IET Sys Biol.

[15] Wan Y, Roy S, Saberi A (2008). A new focus in the science of networks: toward methods for design.. Proc Roy Soc A.

[16] (2006).

[17] Berhe G, Belihu K, Asfaw Y (2007). Seroepidemiological investigation of bovine brucellosis in the extensive cattle production system of Tigray region of Ethiopia.. Int Jour App Res Vet Med.

[18] Smits HL, Cutler SJ (2004). Application of biotechnology to the control and prevention of brucellosis in Africa.. Af Jour Biotech.

[19] (1998).

[20] Rugh W (1995).

[21] Roy S, Saberi A, Stoorvogel A (2007). Toward a control theory for networks, editorial in the Int Jour Rob Nonlin Cont.. Special Issue on Communicating-Agent Networks.

[22] Baillieul J, Antsaklis PJ (2007). Control and communication challenges in networked real-time systems.. Proc IEEE.

[23] Strogatz SH (2001). Exploring complex networks.. Nature.

[24] Watts DJ, Strogatz SH (1998). Collective dynamics of ‘small-world’ networks.. Nature.

[25] Wu CW, Chua LO (1995). Application of Kronecker products to the analysis of systems with uniform linear coupling.. IEEE Trans Circ Sys I.

[26] Roy S, Wan Y, Saberi A (2009).

[27] Wang X, Wan Y, Roy S, Saberi A (2010).

[28] Wilkinson J (1965).

[29] Roy S, Saberi A (2007). Scaling: a canonical design problem for networks.. Int Jour Cont.

[30] Wan Y, Roy S, Saberi A, Stoorvogel A (2010).

[31] Peterson MJ, Grant WE, Davis DS (1992). Bison-brucellosis management: simulation of alternative strategies.. Jour Wild Manage.

[32] Peterson MJ, Grant WE, Davis DS (1991). Simulation of host-parasite interactions within a resource-management framework.. Eco Model.

[33] Unger F, Munstermann S, Goumou A, Apia CN, Konte M (2006). Risk associated with bovine brucellosis in selected study herds and market places in four countries of West Africa..

[34] Ljung L (1987).

[35] Wan Y, Roy S (2009).

[36] Elderd BD, Dukic VM, Dwyer G

